# Interleukin-1β-31C/T and -511T/C Polymorphisms Were Associated with Preeclampsia in Chinese Han Population

**DOI:** 10.1371/journal.pone.0106919

**Published:** 2014-09-15

**Authors:** Xuefeng Wang, Fengli Jiang, Yu Liang, Lina Xu, Hongbo Li, Yali Liu, Shiguo Liu, Yuanhua Ye

**Affiliations:** 1 Prenatal diagnosis center, the Affiliated Hospital of Qingdao University, Qingdao, China; 2 Shandong Provincial Key Laboratory of Metabolic Disease, the Affiliated Hospital of Qingdao University, Qingdao, China; 3 Department of clinical laboratory diagnosis, Medical College, Qingdao University, Qingdao, China; 4 Department of Interventional radiology, the Affiliated Hospital of Qingdao University, Qingdao, China; 5 Department of Respiratory Medicine, Heze Medical College, Heze, China; 6 Department of Interventional radiology, The Songshan Hospital of Medical College, Qingdao University, Qingdao, China; 7 Internal medicine, The Qingdao Fuwai Cardiovascular Hospital, Qingdao, Qingdao, China; CHA University, Republic of Korea

## Abstract

**Objective:**

The purpose of our study is to investigate the relationship between *IL-1β* -31C/T (rs1143627) and -511T/C (rs16944) polymorphisms and the preeclampsia (PE), and analyze the Linkage disequilibrium (LD) and haplotype frequency of the two polymorphism loci.

**Methods:**

Polymorphisms at -31C/T and -511T/C of *IL-1β* were genotyped with the method of polymerase chain reaction-restriction fragment length polymorphism (PCR- RFLP) in 232 PE and 447 control subjects. Genotype and allele frequencies between case-control groups were compared by chi-square(X^2^) tests. Two-point LD and haplotype frequency analyses were done with the software Haploview4.2.

**Results:**

Significant statistical differences were found between PE and control groups regarding genotype and allele frequencies of the two polymorphisms of *IL-1β* (For *IL-1β* -31C/T: X^2^ = 11.478, P = 0.003; For *IL-1β*-511T/C: X^2^ = 9.687, P = 0.008). LD analysis revealed that the IL-1β -31C/T SNP was in high LD with the *IL-1β*-511C/T SNP(D′ = 0.92, r^2^ = 0.79). Both CT and TC haplotypes showed significant differences between case and control groups. Only the plasma level of Prothrombin Time had a significantly statistical difference among TT, CT and CC groups of the preeclamptic two polymorphisms of IL-1β-31C/T and -511T/C (for IL-1β-31C/T, F = 1.644, P = 0.01; F = 1.587, P = 0.016).

**Conclusion:**

Our results revealed *IL-1β* was associated with the PE in Chinese Han population. The CT haplotype may increase the risk of PE, while haplotype TC could be considered as a protective haplotype of PE.

## Introduction

Preeclampsia(PE) is a pregnancy-specific syndrome, characterized by the new onset hypertension and proteinuria after 20^th^ week of gestation. PE occurs in about 5%–7% of all pregnancies in the world, which is a leading cause of the maternal and fetal mortality and morbidity [1]. Although extensive efforts to evaluate the mechanisms and molecules of PE, the underlying pathogenesis of PE has not yet been fully elucidated. Epidemiological research indicated that the PE was associated with the family and genetic control, and inheritance appeared to have a major function in the pathology of this disease [2]. Moreover, PE is a multiple gene disorder, affected by genetic and environmental factors, like interaction between maternal and fetal genes, which were important determinants of maternal disease susceptibility [3]. Therefore, genetic factors cannot be ignored in the pathogenesis of PE.

Despite the etiology of PE remains unknown, a growing evidences indicated that the inflammatory response might explain the development of the PE [4]. It was reported that the normal pregnancy was regarded as a mild inflammatory state [5], while PE was considered to be an exaggerated inflammatory state [5], [6]. Furthermore, PE had been proposed to be a complicated systemic inflammatory response acting in network, which contained not only the endothelium but also the inflammatory immune cells, the clotting and the complementary systems, metabolic and other changes mainly regulated by cytokines [7]. During pregnancy, many cytokines are secreted by immune cells and lymphocytes at the interface of trophoblast and decidua, which mainly mediate and regulate immunity, inflammation and hematopoiesis. Benyo et al indicated that several cytokines had been found to be increased in pregnant women with PE. The serum level of inflammation cytokines, one kind of cytokines, such as *IL-1β*, *IL-2*, *IL-6* and *IFN-γ* had been found to be higher in women with PE than in normotensive pregnant women [8]–[10], which leaded to harmful Th1 immunity, threatening pregnancy by generating cytotoxic factors that injured maternal endothelium, altered steroid hormones biosynthesis and affected other factors which were implicated in trophoblast invasion and maternal spiral artery remodeling [11], [12]. The production of inflammatory cytokines is regulated by the cytokine gene, thus, the cytokines gene polymorphism may play a key role in the development of PE.

IL-1β is a pro-inflammatory cytokines, belonging to the IL-1 system, which is an important role in mammalian reproduction. It had been reported that IL-1β had been implicated in the pathogenesis of PE [13]. Many researchers had found that the plasma level of IL-1β was elevated in preeclamptic women [10], [14]–[16]. And increased placental expression of IL-1β was also observed in several researches [14], [17].

IL-1β is located in 70–110 kb region of chromosome 2q13–21, including 7 exons and 6 introns. At least 20 SNPs have been reported in the region of IL-1β, among which -511 and-31 loci in the promoter region of IL-1β were two important single nucleotide polymorphisms, whose gene polymorphism could influence the gene transcription and lead to the function changes [18], [19]. Moreover, the SNPS −511 and −31 cites have been repeatedly associated with cardiovascular diseases [19]. In the previous studies, Hefler et al, Lachmeijer et al and Lin et al observed the genotypic distribution of the *IL-1β* at position +3954 in exon and the IL-1β-511 C/T polymorphism respectively in Hispanic population, Dutch population and Taiwanese women, similarly, they all found no significantly statistical at the loci between the PE and normal pregnant women [20]–[22]. Mohajertehran et al studied the polymorphism of *IL-1β*(C+3954T) in exon 5, however, no significantly statistical difference was found between the PE and the health pregnant women [23].Currently, the linkage between the -31 loci and PE has not been reported. The aim of our research is to evaluate the association between the two gene polymorphisms of *IL-1β* and PE.

## Material and Methods

### Subjects

The study was approved by the ethics committee of Affiliated Hospital of Qingdao University Medical College. All PE and normal control objects have given written informed consents before participation in the research. 232PE patients and 447normal control pregnant women were recruited from Affiliated Hospital of Qingdao University Medical College and Linyi People's Hospital. The research staffs filled out the questionnaire to all study objects, containing not only the maternal age, gestational age, gravidity, parity and the number of previous abortions but also detailed medical history systemic physical and pelvic examinations, complete blood counts, routine urinary and biochemical analysis, which is beneficial to the statistical analysis of the study results.

The PE was defined as blood pressure of ≥140/90 with proteinuria C≥300 mg in 24 h or ≥2+dipstick after 20 weeks of gestation [24]. The control groups are composed of normal pregnant women, matching with the case group according age(maternal age≥26years). Both case and control pregnant women have no previous history of PE and a systemic disease such as diabetes mellitus, chronic hypertension, chronic renal failure, thyroid function disorder, heart disease or other medical pregnancy complication like premature rupture of membranes, placenta previa, threatened abortion, artificial insemination, twin/multiple pregnancies, premature birth et al. Moreover, the healthy pregnant women were followed up and we selected the normal delivery pregnant people as control group. Both case and control pregnant women have no smoke history.

### Genetic studies

Genomic DNA was extracted from the peripheral venous blood using DNA extraction kit according to standard operation. The amplification primers that we used are as follows: IL-1β -31 locus: forward 5′-AGAAGCTTCCACCAATACTC-3′, Reverse 5′-AGCACCTAGTTGTAAGGAAG -3′; IL-1β -511 locus: forward 5′-TGGCATTGATCTGGTTCATC-3′, Reverse 5′-GTTTAGGAATCTTCCCACTT-3′. All PCR amplification system were performed in 10* µL* volume, including 3.5* µL* ultrapure water, 5* µL* mix, 0.25* µL* forward primers, 0.25* µL* reverse primers and 1* µl* isolated of DNA. The PCR conditions of -511T/C polymorphism of *IL-1β*were initial denaturation at 94°C for 5 min; 35 cycles of denaturation at 94°C for 30 s, annealing at 58°C for 30 s and extension at 72°C for 30 s and final tension at 72°C for 7 min. The annealing temperature of the polymorphism of -31C/T of IL-1β is 57°C. Then the two kinds of PCR fragments was digested by AvaI enzyme and AluI enzyme respectively at 37°C for 24 h in a reaction volume of 10* µL*. Digested products were separated by electrophoresis on a 2.5% agarose gel and visualized by Goodview staining. The expected results of IL-1β-31C/T were: CC: 239 bp, TT: 137 bp, 102 bp, CT: 239 bp,137 bp,102 bp. And The IL-1β-511T/C is digested into 3 expected results: TT showing one band with 518 bp, CC showing two bands with 403 bp and 115 bp, TC showing three bands with518 bp, 403 bp and 115 bp.

### Statistical methods

Statistical analysis of the data was performed with SPSS 17.0 software package. Continuous data variables was described by mean ± standard deviation, and categorical variables was expressed using percentage. Statistical evaluation of patients' data in terms of age, gestational age, number of gravidity, abortion, parity and mean arterial pressure was performed using Student's t-test or Mann-Whitney test. Difference in the distribution of genotype and allele between case-control groups were analyzed by the chi-square statistical, which was used for deviation of genotypic distribution from Hardy-Weinberg equilibrium. Linkage disequilibrium(LD) among polymorphisms of *IL-1β* and the haplotype frequencies were tested using Haploview4.2 software. The One-way ANOVA was used to analyze the laboratory examination of PE among different genotypes. A level of P<0.05 was considered statistical significance.

## Results

### Demographic and clinical characteristics

The demographic and clinical characteristics of the study population were shown in Table 1. The demographics such as age, gravidity and the number of abortions were similar between the PE patients and normal controls (all P>0.05). The gestational age at delivery was found to be significant different among the case and control groups. Furthermore, the birth weight of the neonates in PE group was obviously lower compared with the neonates in the control pregnant women, which had a significantly statistical difference(X^2^ = 9.084, P<0.01). The mean systolic blood pressure of the objects in case and control groups were 146.68±24.38 (mmHg) and 112.53±10.47(mmHg) respectively. The average diastolic blood pressures of the objects in case and control groups were respectively 97.59±18.03 (mmHg) and 74.06±8.52(mmHg). And a significant difference was observed in systolic and diastolic blood pressures between the PE patients and healthy control group (P<0.01).

**Table 1:** Demographic and clinical characteristics of the PE cohort and normal pregnant women.

	PE	control	*X^2^*	P
Maternal age(years)	30.50±5.20	30.43±4.33	0.527	0.598^a^
Gestational age(weeks)	34.50±4.14	38.59±2.12	12.318	P<0.01^a^
Gestational age of at delivery(weeks)	36.62±2.96	39.35±1.47	10.624	P<0.01^a^
gravidity	2.33±1.26	2.30±1.23	0.223	0.824
Number of abortions	0.86±1.03	0.79±0.95	1.015	0.394
Birth weight(g)	2695.08±912.75	3408.47±493.28	9.08	P<0.01^a^
Systolic blood pressure(mmHg)	146.68±24.38	112.53±10.47	16.13	P<0.01^a^
Systolic blood pressure(mmHg)	97.59±18.03	74.06±8.52	15.576	P<0.01^a^

a Mann-Whitney test.

### Genotype distributions and allele frequencies

The genotypic distributions of the two polymorphisms at position −511 and −31 in the promoter region of the IL-1βin case and control groups were all consisted with Hardy-Weinberg equilibrium and had a group representative.

The frequency of IL-1β C-31T polymorphism in PE and normal pregnant women was summarized in Table 2. The polymorphism of -31C/T was significantly associated with the PE (X^2^ = 11.478, P = 0.003). And the percentage of homozygotes (CC) was significantly higher in PE women group than the control group(38.8%vs26.17%). To further analysis the value of OR, it was found that the women with a CC genotype showed more possibility of experiencing PE than those with TT and CT genotypes (X^2^ = 11.476, P = 0.001, OR = 1.788, 95%CI = 1.275–2.506). And the heterozygote polymorphic genotype (CT) appeared to play a protective effect against the risk of PE (X^2^ = 4.151, P = 0.042, OR = 0.715, 95%CI = 0.518–0.988). Moreover, there was a significantly statistical difference of the allele frequency between the PE and healthy pregnant women (X^2^ = 8.904, P = 0.003).

**Table 2:** Distribution the allele frequency and PE susceptibility of the IL-1β -31C/T polymorphism between case and control groups.

	TT	CC	CT	T	C
PE	52(22.41%)	90(38.80%)	90(38.80%)	194(41.81%)	270(58.19%)
control	120(26.85%)	117(26.17%)	210(46.98%)	450(50.34%)	444(49.66%)
*χ* ^2^	1.586	11.476	4.151	8.904	8.904
*P*	0.208	*p*<0.001	0.042	0.003	0.003
*OR*	0.787	1.788	0.715	0.709	1.411
*95%CI*	0.542∼1.143	1.275∼2.506	0.518∼0.988	0.565∼0.889	1.125∼1.769

Another polymorphic analysis data of -511C/T in *IL-1β* studied in our research was displayed in Table 3. Not only the genotypic distribution but also the allele frequency did show a significantly statistical difference among case and control groups (for genotypic distribution X^2^ = 9.687, P = 0.008; for allele frequency X^2^ = 8.557, P = 0.003). As shown in Table 3, the subjects carrying the TT genotype had 1.716 fold risk of the PE compared with the women with CT and CC genotypes(X^2^ = 9.479, P = 0.002, OR = 1.716, 95%CI = 1.215–2.424). The results of allele also showed that the T allele might be a risk factor of the PE(X^2^ = 8.557, P = 0.003, OR = 1.4, 95%CI = 1.117–1.754).

**Table 3:** Distribution the allele frequency and PE susceptibility of the IL-1β-511T/C polymorphism between case and control groups.

	TT	CC	TC	T	C
PE	82(35.34%)	52(22.41%)	98(42.25%)	262(56.47%)	202(43.73%)
control	108(24.16%)	125(27.96%)	214(47.88%)	430(48.10%)	464(51.90%)
*χ* ^2^	9.479	2.441	1.952	8.557	8.557
*P*	0.002	0.208	0.162	0.003	0.003
*OR*	1.716	0.787	0.796	1.4	0.714
*95%CI*	1.215∼2.424	0.542∼1.143	0.578∼1.096	1.117∼1.754	0.570∼0.895

### Linkage disequilibrium and haplotype analysis

To further examine the relationship between *IL-1β* promoter haplotypic structure and the PE, haplotypic groups were constructed using the Haploview4.2 software. The analysis of the LD across the *IL-1β* indicated that the IL-1β-31C/T SNP was in high LD with the IL-1β-511C/T SNP (D′ = 0.92, r^2^ = 0.79) (shown in table 4). Two polymorphisms of −31C/T and −511T/C defined four major haplotypes, which produced the following distribution: 49.1% CT (IL-1β-31T/-511T), 45.5%TC, 3.5% CC, 1.9% TT. Significantly statistical differences were observed in haplotype CT and TC between the case and control groups(X^2^ = 7.63, P = 0.0057; X^2^ = 9.958, P = 0.0016). Analyses demonstrated that individuals with *IL-1β* CT haplotype were significantly more susceptible than individuals without the haplotype. In contrast, carriage of TC haplotype was associated with protection against the PE.

**Table 4:** The distribution of haplotype embracing the two loci, −31 and −511 in the promoter of *IL-1β.*

Haplotype	Freq.	Case	control	X2	P Value
CT	0.491	0.464,	0.543	7.63	0.0057
TC	0.455	0.486,	0.396	9.958	0.0016
CC	0.035	0.033,	0.039	0.35	0.554
TT	0.019	0.017,	0.022	0.355	0.5511

### Comparison of laboratory examination of PE among different genotypes

The analysis of laboratory examination of PE among different genotypes was displayed in table 5. After evaluated all the laboratory tests of the PE, we found that only the Prothrombin Time (PT) plasma level had a significantly statistical difference among TT, CT and CC groups of the PE. For the polymorphism at the position −31 in the promoter region of *IL-1β*, the mean ±SD of the PT were 9.9919±1.62639(s), 10.2140±1.79330(s), 11.1649±1.59619(s) respectively in TT,CC,CT groups of PE. About another polymorphism of IL-1β-511C/T, the mean ±SD of the PT were 10.1078±1.71152, 10.2553±1.81285, 11.0190±1.61771 respectively in CC, TT, CT PE groups. Both the PT level of PE among different genotypes of the two variants of *IL-1β* showed an obvious statistical significance. (All P<0.01).

**Table 5:** The PT plasma level of PE among different genotypes.

genotype	-31C/T(s)	-511C/T(s)
TT	10.21±1.79	10.11±1.71
CC	9.99±1.63	10.26±1.81
CT	11.16±1.60	11.02±1.61
F	1.644	1.587
P	0.01	0.010

## Discussion

IL-1β is a pro-inflammatory cytokine, secreted by monocytes, macrophages and epithelial cells, which implicates in a variety activities. *IL-1β* has previously been found to increase the production of TNF-α, IL-6, HCG, endothelia [10], [17], [25], which were proved to be associated with the development of PE. *IL-1β* may also involve in the oxidative stress linked with PE by stimulating the secretion of other lymphocytotropic cytokines and catabolic enzymes [26]. In addition, *IL-1β* has been considered to be a potential mediator of maternal endothelial dysfunction in PE [27]. Beyond that, it has been shown that *IL-1β* played an important role in abnormal extrarillous trophoblast invasion in PE [28].

In our present study, we investigated the relationship between the -31C/T and the -511T/C polymorphisms of the *IL-1β* and PE. It was worthwhile to note that the two polymorphisms in the promoter region of the *IL-1β* were all associated with the genesis of the PE(all P<0.05). After calculation of the value of OR, the pregnant women bearing the CC genotype revealed a 1.788fold risk of developing the PE at the position −31 in the promoter region of the *IL-1β*(X^2^ = 11.476, P = 0.001, OR = 1.788, 95%CI = 1.275–2.506), while the individuals with TT genotype of the −511 T/C polymorphism of the *IL-1β* were more susceptibility of experiencing the PE(X^2^ = 9.479, P = 0.002, OR = 1.716, 95%CI = 1.215–2.424). Only the CT genotype of the C-31T polymorphism was found to have the effect of protecting the pregnant women from the PE. As for allele, the −31 C allele and −511 T allele were all observed to be associated with a high risk of the PE in the similar manner. However, our research conclusion did not agree with the previous study views. Lin kang et al studied the 102 women with PE and 148 controls and found that there was not any role of the polymorphism of the IL-1β-511T/C in the pathogenesis of PE among Taiwanese population [21]. Moreover, another two experiments also found the similar phenomenon respectively in Hispanic women [20] and in Holland population [22]. The discrepancy may due to the different ethnic groups, the scale of the sample, the population stratification and so on, which needs to be elucidated by further researches.

In addition, we also analyzed the LD and haplotype frequency of the two di-alleic polymorphisms of the *IL-1β*. The two polymorphisms −31and −511 in IL-1β, which were significantly associated with PE, were in high LD (r^2^ = 0.72). Haplotypes constructed using the IL-1β −31and−511 by Haploview4.2 software were significantly related to the PE. The IL-1β promoter haplotypes CT and TC were associated with the development of PE. The haplotype CT may add the risk of PE, while the haplotype TC could be considered as a protective haplotype of PE. However, our present study only studied the two polymorphisms, further study are needed to investigate whether other SNPs involve in the genesis of PE.

But beyond that, the relationship between the laboratory tests and the different genotypes of PE were also investigated. After assessed, we found that only the PT plasma level had a significantly statistical difference among TT, CT and CC groups of the two polymorphisms of *IL-1β* (forIL-1β-31C/T: F = 1.644, P = 0.01; for IL-1β -511T/C: F = 1.587, P = 0.016). Moreover, previous researches had indicated that hematocrit, platelet counts and indices and the Mean platelet volume (MPV) were useful as screening test for early diagnosis of PE [29]–[31]. Basing on the above results, PT level in plasma may be regarded as a marker of the genotype, which needs to be clarified by further studies.

In conclusion, we found evidences that the two polymorphisms at the position −31and −511 in the promoter region of the *IL-1β* were associated with PE in Chinese han population. And the analysis of the LD indicated that the IL-1β-31C/T SNP was in high LD with the IL-1β-511T/C SNP. The IL-1β promoter haplotype CT and TC were associated with the development of PE. The haplotype CT may add the risk of PE, while the haplotype TC could be considered as a protective haplotype of PE. As we all know, PE was regarded as a multifactorial disease, both the environmental risk factors and the genetic factors could influence our research results. Therefore, further genetic and function studies in samples with different ethnicities are needed to validate our finding and find out whether other polymorphisms loci of *IL-1β* participate in the genesis of PE.
